# Retrospective Analysis of Prognostic Factors in 205 Patients with Laryngeal Squamous Cell Carcinoma Who Underwent Surgical Treatment

**DOI:** 10.1371/journal.pone.0060157

**Published:** 2013-04-04

**Authors:** Si-Yi Zhang, Zhong-Ming Lu, Xiao-Ning Luo, Liang-Si Chen, Ping-Jiang Ge, Xin-Han Song, Shao-hua Chen, Yi-Long Wu

**Affiliations:** 1 Department of Otorhinolaryngology, Guangdong General Hospital & Guangdong Academy of Medical Sciences, Guangzhou, Guangdong Province, China; 2 Southern Medical University, Guangzhou, Guangdong Province, China; 3 Guangdong Lung Cancer Institute, Guangdong General Hospital & Guangdong Academy of Medical Sciences, Guangzhou, Guangdong Province, China; Virginia Commonwealth University, United States of America

## Abstract

**Objectives:**

To investigate the most important factors affecting the prognosis of the patients with squamous cell carcinoma (SCC) of the larynx.

**Methods:**

Based on the clinical and follow-up data, 205 patients with SCC of the larynx receiving total laryngectomy, partial laryngectomy, or CO_2_ laser surgery in GuangDong General Hospital were retrospectively analyzed. A survival analysis was performed by the Kaplan-Meier method and a multivariable analysis of prognostic factors was carried out using the Cox proportional hazard model.

**Results:**

Subtypes of carcinoma included 69.8% glottic and 30.2% supraglottic. Most patients were in N0 stage (77.6%), and 22.4% patients were in N1∼N3 stage. Over half of the patients were in T1∼T2 stage (55.1%), 20.0% in T3, and 24.9% in T4. Mean follow-up duration was 49.2 months. The survival rates 1, 2, and 3 years after the surgery were 99.0%, 91.7%, and 81.5%, respectively. The survival rate for those patients with clinical stage IV was significantly lower than for those with clinical stage I and II (*p*<0.001 and *p* = 0.013, respectively). The disease-free progression rates 1, 2, and 3 years after the surgery were 83.9%, 74.6%, and 71.2%, respectively. Futhermore, those patients with a Charlson score of 1 to 2 and ≥3 had higher risk of mortality than those with a Charlson score of 0 (hazard ratios of 1.8 and 2.41 *p* = 0.042 and *p* = 0.008). Multivariable analysis revealed that clinical stage, surgical margin, and comorbidity were significantly associated with both mortality and disease-free progression.

**Conclusion:**

The surgical resection margin, clinical stage, and comorbidity were independent factors affecting the laryngeal cancer prognosis. The survival rates were lower for patients with advanced laryngeal cancer, positive surgical margins, or severe comorbidity, suggesting the importance of early diagnosis, early treatment, negative surgical margins, and conditions of comorbidity.

## Introduction

Laryngeal cancer is a malignant disease with high incidence and it accounts for about 2.4% of all newly diagnosed malignancies worldwide each year [1].With the continuous industrialization in China, the prevalence has gradually increased in this country to about 2 to 4/100, 000 [2]. Laryngeal cancer is the second most common malignant tumor of the head and neck tumors, and it often occurs in middle-aged and elderly men. It is generally believed that the occurrence of laryngeal cancer may be related to alcohol consumption, tobacco use, air pollution, sex hormones, and occupational factors [3]–[5]. In recent years, the survival rate of laryngocarcinoma patients has been demonstrating a decreasing trend (from 57.1% to 51.9%) although the survival of patients with other cancers has been prolonged to different extents [6]. Elucidating the survival rate of laryngocarcinoma patients will be beneficial in determining the survival trend.

Understanding the prognostic factors for patients after laryngeal cancer surgery may reverse the trend toward a decreased rate of survival and greatly help to improve both the treatment strategies and therefore the survival rate of patients. However, the prognostic factors are currently still controversial [6], and they require further study.

Surgical treatment is the main means of treatment for laryngeal cancer and its efficacy is superior to the single non-surgical treatment [8]. For example, patients with stage IV disease experienced significantly better survival with surgery (49%) than with chemoradiation (21%) or radiation alone (14%) (*p*<.0001). As China has a large population, and the development in different regions varies widely, the surgical treatment of laryngocarcinoma has not been standardized in a lot of primary settings. Some aspects of treatment are in need of improvement. Thus, investigating the prognostic factors of laryngocarcinoma is undoubtedly crucial for raising the therapeutic efficacy of treatments for this disease.

In this study, we retrospectively analyzed the prognostic factors for patients with laryngeal squamous cell carcinoma who were treated at our hospital from 2003 to 2008 including age, gender, smoking, drinking, staging, classification, thyroid cartilage invasion, surgical margins, pathological differentiation, postoperative radiotherapy, and comorbidity. We studied the effects of those factors on the prognosis of patients with laryngeal cancer. This study should help to identify the prognostic factors for patient survival after laryngeal cancer surgery and may provide important insights that can improve the treatment strategies.

## Methods

Patients with laryngeal cancer who were admitted to and treated at our hospital from January 2003 to November 2008 (their squamous cell carcinoma was confirmed by pathological biopsy) were included. Staging of laryngeal cancer was based on the UICC-TNM 2002 criteria [9], [10]. The duration of follow-up was >36 months in all patients. Patients with known distant metastases or non-squamous cell malignancies before treatment and patients who could not complete the treatment or were lost to follow-up were excluded.

Surgical indications: Patients with biopsy-proven laryngeal squamous cell carcinoma as confirmed by pathology, without distant metastasis, and who understood and agreed to accept the surgery.

For the selected patients, prognostic factors of laryngeal squamous cell carcinoma including age, gender, smoking, alcohol consumption, stage, classification (anatomical site), thyroid cartilage invasion, surgical margins, pathological differentiation, postoperative radiotherapy, and comorbidity were collected. The extent of comorbidities was determined using the Charlson comorbidity index (CI) [11].

The criteria for determining disease-free state and disease progression were as follow: fiberoptic laryngoscopy, electric laryngoscopy and imaging examinations (CT, MRI, PET, chest X ray, liver ultrasonography) were performed to evaluate suspicious lesions. When suspicious lesions were observed, biopsies with subsequent pathological examination were performed to determine if laryngeal recurrence and/or regional lymphatic or distant metastasis had occurred. A disease-free state was defined as the absence of cancer demonstrated by imaging examinations, and (if necessary) pathological examination following biopsy. Post-operative complications were not included the definition of disease state, which only referred to the presence, recurrence or metastasis of cancer.

Patients were followed up every 3 months for survival status, disease progression, time of death (if applicable), and post-operative complications (such as pharyngeal fistula, pneumonia, surgical wound infection, laryngeal granuloma, etc.).

This study was approved by the Research Ethics Committee of Guangdong General Hospital & Guangdong Academy of Medical Sciences in written form and all participants provided written informed consent.

### Statistical Analysis

Age was presented as mean and standard deviation, and other category variables were presented as number and percentages. The overall survival (OS) rate and disease progression-free (DPF) rate were estimated using the Kaplan-Meier curves. The comparisons of the Kaplan-Meier curves by clinical stage and surgical margin were performed by the log-rank test. The Cox proportional hazard ratio models were performed to evaluate the independent factors of mortality and disease-progression (recurrence and metastasis) free rate. The observed significant factors in the univariable Cox proportional hazard ratio models were stepwise entered into the multivariable Cox proportional hazard ratio model, except for T and N stages that were excluded due to multicollinearity. A *p* value <0.05 was considered as statistically significant. All statistical hypothesis tests were two-sided. Statistical analyses were performed using SPSS15.0 (SPSS Inc, Chicago, IL, USA).

## Results

### Baseline Characteristics

During the study period from January 2003 to November 2008, 301 patients with laryngeal carcinoma were hospitalized, with 96 of them being excluded from the study according to the exclusion criteria. The remaining 205 patients were analyzed, including 197 (96.1%) males and 8 females (3.9%), and their mean age was 61.8 years (Table 1). Over 60% of the patients had a smoking index of 20 pack-year or more, and 31.2% patients drank frequently. The types of carcinoma included 69.8% glottic and 30.2% supraglottic. Most of the patients were in N0 stage (77.6%), and 22.4% patients were in N1 to N3 stage. Over half of the patients were in T1 to T2 stage (55.1%), 20.0% in T3, and 24.9% in T4. Nearly 30% of the patients were at clinical stage I, and more than 30%were at clinical stage IV. Over half of the patients had a Charlson score of 0, 27.3% had a Charlson score of 1–2, and 17.1% had a Charlson score of 3 or above. About one-fourth of the patients had thyroid cartilage invasion, and 19.5% and 46.3% patients had low and moderate pathological differentiation, respectively.

**Table 1:** Summary of baseline characteristics.

			N = 205
Age (year)			61.8±10.6
Gender		Male	197 (96.1)
		Female	8 (3.9)
Smoking index		<20 pack-year	80 (39.0)
		≥20 pack-year	125 (61.0)
Drinking		Frequently	64 (31.2)
		None or occasionally	141 (68.8)
Type		Supraglottic	62 (30.2)
		Glottic	143 (69.8)
Clinical stage		I	60 (29.3)
		II	42 (20.5)
		III	39 (19.0)
		IV	64 (31.2)
T stage		T1∼T2	113 (55.1)
		T3	41 (20.0)
		T4	51 (24.9)
N stage		N0	159 (77.6)
		N1∼N3	46 (22.4)
Charlson score		0	114 (55.6)
		1–2	56 (27.3)
		≥3	35 (17.1)
Thyroid cartilage invasion			51 (24.9)
Pathological differentiation	Poorly differentiated		40 (19.5)
	Moderately differentiated		95 (46.3)
	Highly differentiated		70 (34.1)

Smoking index = number of cigarettes daily×smoking years; Drinking: “none or occasionally” means absolutely no alcohol consumption or once weekly, with each drink less than 250 ml of beer or 62.5 g of hard alcohol;“frequently” means alcohol consumption at least twice weekly, with each drink comprising at least one cup of beer or 62.5 g of hard alcohol.

### Treatments and Complications

Surgical treatment of the primary lesion included total laryngectomy (71 cases, 34.6%), partial laryngectomy or CO_2_ laser microlaryngoscopic resection (134 cases, 65.4%). Total laryngectomy was indicated for patients with tumor grade T3 or above, while partial laryngectomy and laser treatment were reserved for tumor grades less than T3, with laser treatment only being routinely performed after the year 2000. The ipsilateral selected area (areas II-IV) neck dissection was adopted for patients with local cN0 advanced cancer and risk of cervical lymph node metastasis. Bilateral selective neck dissection was adopted for patients with supraglottic cancer. Some patients received postoperative radiotherapy or chemotherapy. Seventeen patients (8.3%) underwent postoperative radiotherapy (XRT): patients with metastasis to more than 3 pathologically confirmed cervical lymph nodes, capsule invasion, T4 lesions, peripheral nerve and vascular invasion. Eight patients (3.9%) received chemotherapy, and the chemotherapy drugs included Cisplatin with or without 5–fluorouracil, and Taxol.

Thirty-one (15.1%) patients had a positive surgical margin (Table 2). Seventeen and eight patients were treated with adjuvant radiotherapy and chemotherapy, respectively. The chemotherapeutic regimens included 2 TPF (Pacilitaxel+Cisplatin +5-fluorouracil), 5 PF (Cisplatin+5-fluorouracil), and 1 cisplatin. Complications occurred in 32 patients, with the two most frequent complications being laryngeal granuloma (n = 16) and pharyngeal fistula (n = 8).

**Table 2:** Summary of the treatments and complications after the surgery.

		N = 205
Surgical margin	Positive	31 (15.1)
	Negative	174 (84.9)
Radiotherapy		17 (8.3)
Chemotherapy		8 (3.9)
Chemotherapeutic drugs		
TPF: Pacilitaxel, Cisplatin, and 5-fluorouracil		2 (1.0)
PF: Cisplatin and 5-fluorouracil		5 (2.4)
P: Cisplatin		1 (0.5)
Complications		32 (15.6)
Laryngeal granuloma		16 (7.8)
Pharyngeal fistula		8 (3.9)
Hemorrhage		3 (1.5)
Pneumonitis		3 (1.5)
Aspiration pneumonitis		1 (0.5)
Wound infection		1 (0.5)
Laryngostenosis		1 (0.5)

### Overall Survival and Disease Progression-free Rates

**Figure 1:**
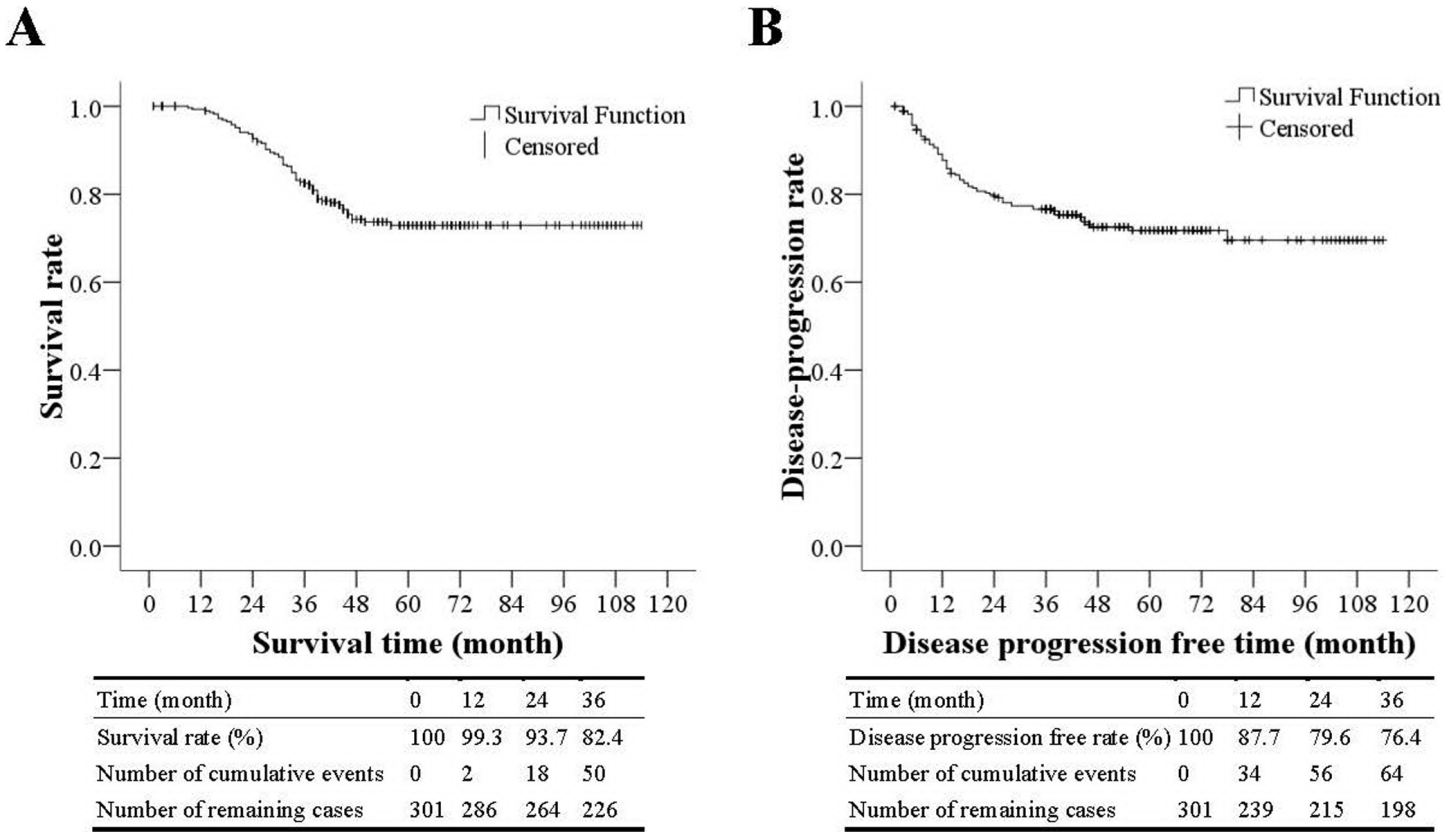
Kaplan-Meier survival curves for overall survival and disease progression-free rate.

All patients were followed for at least 36 months, and the mean follow-up duration was 49.2 months. The survival rates 1, 2, and 3 years after the surgery were 99.0%, 91.7%, and 81.5%, respectively (Figure 1). The disease-free progression rates 1, 2, and 3 years after the surgery were 83.9%, 74.6%, and 71.2%, respectively.

### The Influence Factors of Mortality

In the univariable analysis, eight variables were significantly associated with mortality, including clinical stage, T stage, N stage, thyroid cartilage invasion, carcinoma type, Charlson score, surgical margin, and pathological differentiation (Table 3). T stage and N stage were first excluded from the multivariable analyses due to multicollinearity, and the other six variables were stepwise entered into the multivariable Cox proportional hazard model by the forward conditional method. After model selection, clinical stage, Charlson score, and surgical margin showed an independent influence on the occurrence of mortality. After controlling for each of the three variables, those with clinical stage IV had higher risk of mortality than those with stage I (hazard ratio of 6.37, *p*<0.001); those with Charlson score in 1–2 and ≥3 had higher risk of mortality than those with Charlson score of 0 (hazard ratios of 2.69 and 3.6, *p* = 0.004 and *p*<0.001); those with positive surgical margin had higher risk of mortality than those with negative surgical margin (hazard ratio of 3.83, *p*<0.001).

**Table 3:** Univariable and multivariable Cox proportional hazard models of mortality.

		Univariable analysis				Multivariable analysis	
		HR (95% CI)		*p*-value	HR (95% CI)		*p*-value
Age (year)		1.02 (0.99, 1.05)		0.119			
Gender	Female	0.45 (0.06, 3.28)	0.434				
	Male	Reference					
Clinical stage	I	Reference				Reference	
	II	1.74 (0.63, 4.81)		0.283	1.69 (0.61, 4.67)		0.309
	III	2.32 (0.88, 6.10)		0.088	2.15 (0.81, 5.71)		0.125
	IV	4.74 (2.06, 10.95)		<0.001*	6.37 (2.70, 15.01)		<0.001*
T stage	T1∼T2	Reference					
	T3	2.32 (1.16, 4.63)	0.017*				
	T4	2.66 (1.39, 5.07)	0.003*				
N stage	N1∼N3	2.58 (1.46, 4.55)	0.001*				
	N0	Reference					
Thyroid cartilage invasion		2.02 (1.13, 3.58)	0.017*				
Type	Supraglottic	2.52 (1.45, 4.37)	0.001*				
	Glottic	Reference					
Smoking index	≥20 pack-year	1.42 (0.78, 2.56)	0.248				
	<20 pack-year	Reference					
Drinking	Frequently	1.35 (0.77, 2.38)	0.298				
	None or occasionally	Reference					
Charlson score	0	Reference					
	1–2	2.00 (1.05, 3.81)	0.036*		2.69 (1.38, 5.24)		0.004*
	≥3	2.68 (1.35, 5.31)	0.005*			3.60 (1.77, 7.33)	<0.001*
Surgical margin	Positive	3.33 (1.86, 5.96)	<0.001*			3.83 (2.08, 7.05)	<0.001*
	Negative	Reference				Reference	
Pathological differentiation	Highly differentiated	Reference					
	Moderately differentiated	1.72 (0.87, 3.43)	0.121				
	Poorly differentiated	2.53 (1.17, 5.47)	0.019*				
Post-surgery radiotherapy		2.07 (0.93, 4.61)	0.074				

T stage and N stage is excluded from the multivariable analysis due to multicollinearity.

**Figure 2:**
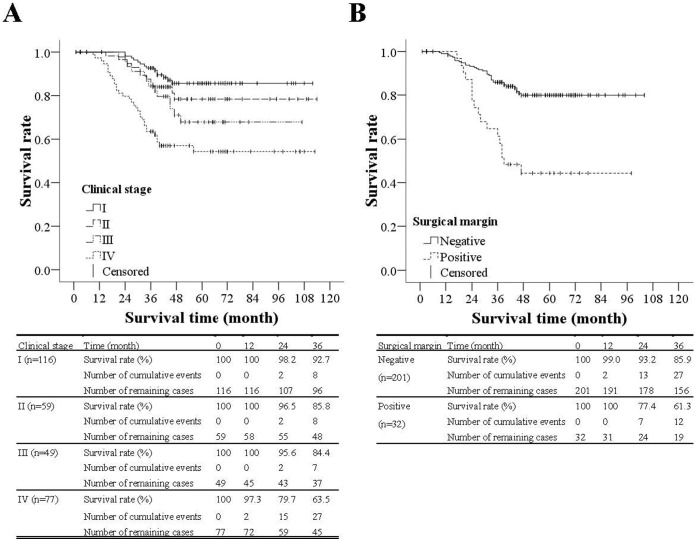
Kaplan-Meier curves for overall survival rate by clinical stage and surgical margin. (A)The survival rate for those with clinical stage IV tumors was significantly lower than those with clinical stage I and II, *p*<0.001 and *p* = 0.013 respectively. (B) The survival rate for those with a positive surgical margin was significantly lower than those with negative surgical margin, *p*<0.001.

Similar results regarding clinical stage and surgical margin in the above multivariable analysis were also shown in the Kaplan-Meier curves for the survival rate (Figure 2). For the patients with clinical stage I tumors, the survival rates 1, 2, and 3 years after the surgery were 100%, 96.7%, and 93.3%, respectively. For clinical stage II tumors, the survival rates 1, 2, and 3 years after the surgery were 100%, 95.2%, and 88.1%, respectively. For clinical stage III, the survival rates during the same timeframe after the surgery were 100%, 94.9%, and 84.6%, respectively. For the patients with clinical stage IV tumors, the survival rates1, 2, and 3 years after the surgery were 96.9%, 78.1%, and 64.1%, respectively. The survival rate for those with clinical stage IV was significantly lower than those with clinical stage I and II (*p*<0.001 and *p* = 0.013, respectively). For the patients whose surgical margins were negative, the survival rates 1, 2, and 3 years after the surgery were 98.9%, 92.5%, and 85.1%, respectively; for the patients whose surgical margins were positive, the survival rates during the same time points after the surgery were 100%, 77.4%, and 61.3%, respectively. The survival rate for those with positive a surgical margin was significantly lower than those with negative surgical margin (*p*<0.001). An additional multivariable analysis excluding clinical stage due to multicollinearity (as in Model II) was performed (Table S1). Based on the additional ST analysis, T and N stages were both independently associated with mortality.

### The Influence Factors of Disease-progression

T stage and N stage were first excluded from the multivariable analyses due to multicollinearity, and the other six variables were entered stepwise into the multivariable Cox proportional hazard model by the forward conditional method. After model selection, only clinical stage, Charlson score, surgical margin, and post-surgery radiotherapy showed an independent influence on the disease progression (Table 4). After controlling for each of these three variables, those with clinical stage IV had a higher risk of disease progression than those with stage I, with a hazard ratio of 3.25 (*p*<0.001); those patients with a Charlson score of 1 to 2 and ≥3 had higher risk of mortality than those with a Charlson score of 0 (hazard ratios of 1.8 and 2.41 *p* = 0.042 and *p* = 0.008); those with a positive surgical margin had higher risk of disease progression than those with negative surgical margin, with a hazard ratio of 2.32 (*p* = 0. 004); those with post-surgery radiotherapy had higher risk of disease progression than those without post-surgery radiotherapy, with a hazard ratio of 2.5 (*p* = 0.011).

**Table 4:** Univariable and multivariable Cox proportional hazard models of disease progression.

		Univariable analysis		Multivariable analysis			
		HR (95% CI)	*p*-value	HR (95% CI)		*p*-value	
Age (year)		1.01 (0.98, 1.03)	0.502				
Gender	Female	0.80 (0.20, 3.26)	0.752				
	Male	Reference					
Clinical stage	I	Reference		Reference			
	II	1.06 (0.47, 2.39)	0.885	0.97 (0.41, 2.28)		0.948	
	III	1.36 (0.63, 2.94)	0.435	1.45 (0.66, 3.20)		0.353	
	IV	2.61 (1.38, 4.94)	0.003*	3.25 (1.68, 6.31)		<0.001*	
T stage	T1∼T2	Reference					
	T3	1.79 (0.98, 3.28)	0.058				
	T4	1.97 (1.12, 3.48)	0.019*				
N stage	N1∼N3	1.99 (1.19, 3.34)	0.009*				
	N0	Reference					
Thyroid cartilage invasion		1.64 (0.98, 2.76)	0.061				
Type	Supraglottic	1.98 (1.21, 3.22)	0.006*				
	Glottic	Reference					
Smoking index	≥400	0.99 (0.60, 1.63)	0.975				
	<400	Reference					
Drinking	Frequently	1.23 (0.74, 2.03)	0.431				
	No drinking or occasionally	Reference					
Charlson score	0	Reference					
	1–2	1.65 (0.95, 2.87)	0.077	1.80 (1.02, 3.16)		0.042*	
	≥3	1.88 (1.01, 3.50)	0.048*	0.008*			
Surgical margin	Positive	2.55 (1.48, 4.39)	<0.001*	2.32 (1.32, 4.09)		0.004*	
	Negative	Reference		Reference			
Pathological differentiation	Highly differentiated	Reference					
	Moderately differentiated	1.75 (0.95, 3.23)	0.072				
	Poorly differentiated	2.47 (1.24, 4.90)	0.010*				
Post-surgery radiotherapy		2.83 (1.48, 5.42)	0.002*	2.50 (1.24, 5.04)		0.011*	

T stage and N stage were excluded from the multivariable analysis due to multicollinearity.

**Figure 3:**
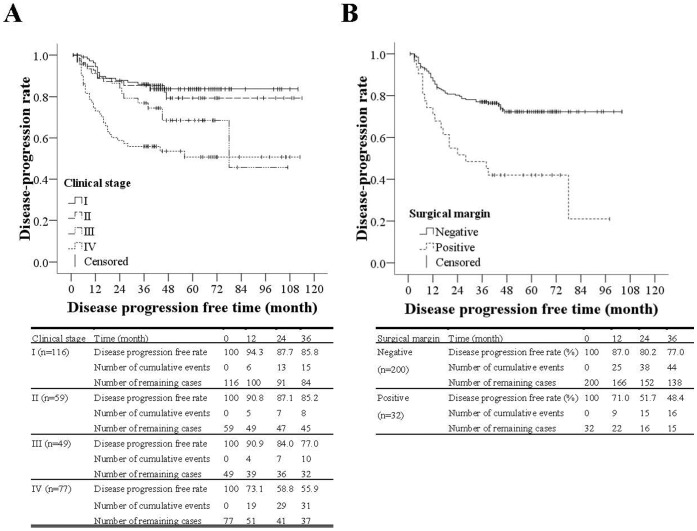
Kaplan-Meier curves for disease progression-free rate by clinical stage and surgical margin. (A)The disease progression-free rate for those with clinical stage IV was significantly lower than those with clinical stage I and II disease, *p* = 0.003 and *p* = 0.014 respectively. (B)The disease progression-free rate for those with positive surgical margin was significantly lower than those with negative surgical margin, *p*<0.001.

Similar results of clinical stage and surgical margin in the above multivariable analysis were also shown in the Kaplan-Meier curves for the disease progression-free rate (Figure 3). For the patients with clinical stage I carcinomas, the disease progression-free rates in 1, 2, and 3 years after the surgery were 93.3%, 83.3%, and 80.2%, respectively. For clinical stage II, the disease progression-free rates during the same time points were 85.7%, 83.3%, and 81.0%, respectively. For clinical stage III tumors, the disease progression-free rates in 1, 2, and 3 years after the surgery were 89.7%, 82.1%, and 76.9%, respectively. For the patients with clinical stage IV carcinomas, the disease progression-free rates during the same time points were 70.3%, 56.3%, and 53.1%, respectively. The disease progression-free rate for those with clinical stage IV was significantly lower than those with clinical stage I and II disease (*p* = 0.003 and *p* = 0.014, respectively) (Figure 3). For the patients with negative surgical margin, the disease progression-free rates in 1, 2, and 3 years after the surgery were 86.2%, 78.7%, and 75.3%, respectively; for the patients with positive surgical margin, the disease progression-free rates during the same time points were 71.0%, 51.6%, and 48.4%, respectively. The disease progression-free rate for those with positive surgical margin was significantly lower than those with negative surgical margin (*p*<0.001). An additional multivariable analysis excluding clinical stage due to multicollinearity (as in Model II) was performed (Table S2). Only T stages were independently associated with disease progression.

## Discussion

Although the survival rates of patients with early-stage laryngeal cancer are high, the survival rates of patients with advanced laryngeal cancer are still relatively low. The survival rate of early-stage laryngeal cancer (Stages I and II) is estimated at 73% to 92% and only 35% to 64% for late-stage laryngeal cancer (Stage III and IV) [6], [8], [10]. Our results showed that the three-year overall survival rate was 81.5%, which is somewhat higher than that observed in several reports in the literature [7], [10], [12]. The possible reasons are that there were more patients with early- and mid-stage laryngeal cancer in our study, and more patients with laryngeal cancer were diagnosed and treated early because the fiber laryngoscope, electronic laryngoscope, CT, or MR were widely used. Therefore, the health status awareness for these patients was improved. Other reasons may include that majority of our patients had glottic laryngeal cancer with more favorable prognosis compared to supraglottic laryngeal cancer, the laryngeal surgery has become increasingly standardized, negative margins are ensured as much as possible during surgery, a postoperative regular follow-up system was established and implemented, and the prognostic factors have been studied in-depth and applied in clinical intervention.

3.1 Multi-factor analysis showed that only surgical resection margin and clinical staging had an impact on efficacy.

The surgical resection margin has a very important impact on prognosis [13], [14]. Our study results also indicated that patients with positive surgical margins had significantly reduced survival rates. A positive surgical resection margin indicates residual tumor cells after surgery, leading to increased risk of local recurrence or cervical lymph node metastasis, which can be the root cause of future recurrence and impaired survival rate. This suggests that it is desirable to achieve a negative surgical margin in order to improve the patient survival rate. After surgical resection, the deep positive tissue at the margin could not be detected early when local tumor recurrence occurs. Even if the patients are followed up closely, the risk of paraglottic space invasion still exists. Therefore, we suggest that patients with positive margins should receive early radiotherapy.

Many studies suggest that among all the prognostic factors for patients with laryngeal cancer, cervical lymph node metastasis is the most important [15], [16]. Jose et al. [17] showed that the 5-year survival rate decreased by about 50% in patients with cervical lymph node metastasis. Another study reported that the hazard ratio of lymph node metastasis for worsening disease-specific survival was 3.92 [16]. The univariable analysis of the current study showed that T stage, N stage, and clinical stage significantly affected the prognosis of patients with laryngeal cancer, and clinical stage independently influenced the survival rate. These results might appear to lessen the effects of cervical lymph node metastasis and T stage and obscure the important influence of cervical lymph node metastasis. Some studies suggest that the lower survival rate of patients with advanced laryngeal squamous cell carcinoma may be due to cervical lymph node metastasis and surgical margin status [13]. The clinical stage comprises the effects of T and N stages, which reflects the prominent impact of local invasion of laryngeal tumors and cervical lymph node metastasis on prognosis comprehensively. Positive margins occur more frequently in patients with advanced laryngeal cancer. Therefore, although positive margins have important prognostic value, the importance of cervical lymph node metastasis for predicting outcome should not be neglected.

There is evidence showing that comorbidity is an important factor influencing the survival of laryngocarcinoma patients in western countries. However, to the best of our knowledge, no studies have reported the influence of comorbidities on the prognosis of laryngocarcinoma in Asian countries. The current study is the first to confirm that comorbidities were also independent factors affecting the prognosis of overall and disease free survival of patients with laryngocarcinoma after surgical intervention, and the value of comorbidities in laryngocarcinoma was comparable to that of clinical stages. In the present study, the Charlson comorbidity index (CI) was used to determine the extent of comorbidities because it presents weighted score of each comorbidity that influences the survival of cancer patients [11]. The Charlson comorbidity index has been validated to be applicable in the evaluation of head and neck cancer [18]. Our results revealed that CI was a useful indicator and should be widely applied in the evaluation of comorbidities.

### 3.2 Influences of a Variety of Other Factors

Whether age affects prognosis is still controversial. In a multivariable analysis, Ramroth et al. [19] showed that age was the most influential factor affecting prognosis of patients with newly diagnosed laryngeal cancer (hazard ratio = 1.5). Du and colleagues [7] found that age was a prognostic factor in the univariable analysis, but it was not an independent prognostic factor. Our study, supports the results of Du’s study. Our results demonstrate that although the survival rate of the elderly was poorer than that of the younger patients, the difference was not statistically different in the multivariable analysis.

There are few lymph vessels in the glottic area, therefore, the glottic carcinoma confined to the vocal cord is not susceptible to cervical lymph node metastasis. On the other hand, in the early stages supraglottic carcinoma usually presents with occult symptoms with richly distributed surrounding lymph vessels. As a result, supraglottic carcinoma is susceptible to cervical lymph node metastasis, especially with occult lymph node metastasis that makes early diagnosis difficult and generally predicts poorer prognosis [20]. In the current study, univariable analysis showed supraglottic carcinoma to carry a poorer prognosis than glottic carcinoma. In addition, most of advanced laryngocarcinoma (stage III/IV) was found to be associated with supraglottic carcinoma. Thus, although multivariable analysis revealed that supraglottic carcinoma was not an independent factor affecting the prognosis of laryngocarcinoma, we still should not neglect its important influence on the prognosis of laryngocarcinoma.

Other studies have found that the pathological stage of tumors is another important prognostic factor for patients with laryngeal cancer [21], [22]. Our results suggested that with the progression of dedifferentiation, the prognosis tends to worsen. However, this trend was only found to be statistically significant in the univariable analysis but not in multivariable analysis. This may have been due to the limited case number in this study. Advanced clinical stage was significantly associated with supraglottic cancers, with 75.8% vs. 39.2% having supraglottic and glottic cancers, respectively (Table S3). Conversely, positive surgical margins were not significantly associated with either cancer.

Currently, many studies have found that postoperative radiotherapy could not improve the prognosis of patients with laryngeal cancer [23], [24]. This observation is also supported by our study, where the addition of postoperative radiotherapy did not improve patients’ prognosis in terms of mortality. Moreover, we found that post-operative radiotherapy was associated with greater likelihood of having poorer disease free progression rate. We think the possible reasons might be the more advanced clinical stage of the patients in the postoperative radiotherapy group, and their highly or moderately differentiated laryngeal cancer, which is not sensitive to radiotherapy. This also suggests the importance of surgery for eradicating these tumors. Regarding post-operative chemotherapy, as only eight patients received the treatment, we did not perform a statistical analysis.

Studies have reported that long-term smoking is a risk factor for laryngeal cancer [12], [25] and that smoking cessation can prevent up to 90% of new cases of laryngeal cancer [26]. Some studies suggest that environmental tobacco smoke (ETS) and smokeless tobacco are also risk factors of laryngeal cancer [27], [28]. Moreover, case-control studies have shown that alcohol intake is an independent factor of laryngeal cancer progression, in addition to having well-known synergistic effects with smoking [22], [29]. The impact of the bad health habits such as alcohol consumption and tobacco use on laryngeal cancer prognosis is still highly controversial, and out results showed that these factors did not cause an effect that was statistically significant. We believe that the actual impact of smoking on the laryngeal cancer prognosis and the mechanisms remain to be confirmed by large-sample prospective studies.

Due to the retrospective design and small sample size, this study had limitations to its utility for basing conclusions. The clinical data from these patients did not include the occupational information, therefore, we could not measure the impact of possible exposure to certain carcinogens at work on the laryngeal cancer incidence and prognosis. That factor may have influenced our study findings to a certain extent.

In summary, surgical resection margin clinical stage, and comorbidity are independent factors affecting laryngeal cancer prognosis. The survival rates are lower in patients with advanced laryngeal cancer or positive surgical margins or severe comorbidity, suggesting the importance of early diagnosis, early treatment, negative surgical margins, and control of comorbidity. In order to improve the survival rates of patients with laryngeal cancer, the importance of early detection of tumors and the need to pay closer attention to surgical margins should be emphasized in the diagnosis and treatment of laryngeal cancer in the future.

## Supporting Information

Table S1 **Multivariable analysis for mortality.** An additional multivariable analysis excluding clinical stage due to multicollinearity (as in Model II) was performed. Based on the additional ST analysis, T and N stages were both independently associated with mortality. Model I: T stage and N stage is excluded due to multicollinearity. Model II: clinical stage is excluded due to multicollinearity.
(DOC)

Table S2 **Multivariable analysis for disease progression.** An additional multivariable analysis excluding clinical stage due to multicollinearity (as in Model II) was performed. Only T stages were independently associated with disease progression. Model I: T stage and N stage is excluded due to multicollinearity. Model II: clinical stage is excluded due to multicollinearity.
(DOC)

Table S3 **Clinical stages and locations.** Advanced clinical stage was significantly associated with supraglottic cancers, with 75.8% vs. 39.2% having supraglottic and glottic cancers, respectively. Conversely, positive surgical margins were not significantly associated with either cancer.
(DOC)
