# Correction: Retrospective Analysis of Prognostic Factors in 205 Patients with Laryngeal Squamous Cell Carcinoma Who Underwent Surgical Treatment

**DOI:** 10.1371/annotation/9c5dbeb2-9990-464e-a05d-72d329f11647

**Published:** 2013-12-16

**Authors:** Si-Yi Zhang, Zhong-Ming Lu, Xiao-Ning Luo, Liang-Si Chen, Ping-Jiang Ge, Xin-Han Song, Shao-hua Chen, Yi-Long Wu

Several errors are present in Figures 2 and 3. Please find the corrected versions of the figures here:

Figure 2: http://plosone.org/corrections/pone.0060157.g002.cn.tif

Figure 3: http://plosone.org/corrections/pone.0060157.g003.cn.tif

