# Supplementary material for: Retrospective Analysis of Prognostic Factors in 205 Patients with Laryngeal Squamous Cell Carcinoma Who Underwent Surgical Treatment
Source: PLoS One. 2013 Apr 4;8(4):e60157. doi: 10.1371/journal.pone.0060157 (PMC3617169; doi:10.1371/journal.pone.0060157)
Supplement: Table S1 — Multivariable analysis for mortality. An additional multivariable analysis excluding clinical stage due to multicollinearity (as in Model II) was performed. Based on the additional ST analysis, T and N stages were both independently associated with mortality. Model I: T stage and N stage is excluded due to multicollinearity. Model II: clinical stage is excluded due to multicollinearity. (DOC) [file pone.0060157.s001.doc]

Table S1. Multivariable analysis for mortality.

|  | | Model I | | Model II | |
| --- | --- | --- | --- | --- | --- |
|  |  | HR (95% CI) | *p*-value | HR (95% CI) | *p*-value |
| Clinical stage | I | Reference |  |  |  |
|  | II | 1.69 (0.61, 4.67) | 0.309 |  |  |
|  | III | 2.15 (0.81, 5.71) | 0.125 |  |  |
|  | IV | 6.37 (2.70, 15.01) | <0.001* |  |  |
| T stage | T1~T2 |  |  | Reference |  |
|  | T3 |  |  | 1.90 (0.92, 3.90) | 0.081 |
|  | T4 |  |  | 2.75 (1.33, 5.68) | 0.006* |
| N stage | N1~N3 |  |  | 1.88 (1.00, 3.53) | 0.049* |
|  | N0 |  |  | Reference |  |
| Charlson score | 0 | Reference |  | Reference |  |
|  | 1-2 | 2.69 (1.38, 5.24) | 0.004* | 2.35 (1.20, 4.61) | 0.012* |
|  | ≥3 | 3.60 (1.77, 7.33) | <0.001* | 3.01 (1.50, 6.03) | 0.002* |
| Surgical margin | Positive | 3.83 (2.08, 7.05) | <0.001* | 3.68 (1.99, 6.81) | <0.001* |
|  | Negative |  |  |  |  |

Model I: T stage and N stage is excluded due to multicollinearity. Model II: clinical stage is excluded due to multicollinearity.
