# Supplementary material for: Retrospective Analysis of Prognostic Factors in 205 Patients with Laryngeal Squamous Cell Carcinoma Who Underwent Surgical Treatment
Source: PLoS One. 2013 Apr 4;8(4):e60157. doi: 10.1371/journal.pone.0060157 (PMC3617169; doi:10.1371/journal.pone.0060157)
Supplement: Table S2 — Multivariable analysis for disease progression. An additional multivariable analysis excluding clinical stage due to multicollinearity (as in Model II) was performed. Only T stages were independently associated with disease progression. Model I: T stage and N stage is excluded due to multicollinearity. Model II: clinical stage is excluded due to multicollinearity. (DOC) [file pone.0060157.s002.doc]

Table S2. Multivariable analysis for disease progression.

|  | | Model I | | Model II | |
| --- | --- | --- | --- | --- | --- |
|  |  | HR (95% CI) | *p*-value | HR (95% CI) | *p*-value |
| Clinical stage | I | Reference |  |  |  |
|  | II | 0.97 (0.41, 2.28) | 0.948 |  |  |
|  | III | 1.45 (0.66, 3.20) | 0.353 |  |  |
|  | IV | 3.25 (1.68, 6.31) | <0.001* |  |  |
| T stage | T1~T2 |  |  | Reference |  |
|  | T3 |  |  | 1.55 (0.81, 2.97) | 0.182 |
|  | T4 |  |  | 2.12 (1.12, 4.02) | 0.022* |
| N stage | N1~N3 |  |  | 1.57 (0.88, 2.79) | 0.125 |
|  | N0 |  |  | Reference |  |
| Charlson score | 0 | Reference |  | Reference |  |
|  | 1-2 | 1.80 (1.02, 3.16) | 0.042* | 1.65 (0.94, 2.91) | 0.082 |
|  | ≥3 | 2.41 (1.26, 4.63) | 0.008* | 2.15 (1.13, 4.08) | 0.020* |
| Surgical margin | Positive | 2.32 (1.32, 4.09) | 0.004* | 2.35 (1.33, 4.15) | 0.003* |
|  | Negative | Reference |  | Reference |  |
| Post-surgery radiotherapy | | 2.50 (1.24, 5.04) | 0.011* | 2.28 (1.11, 4.70) | 0.025* |

Model I: T stage and N stage is excluded due to multicollinearity. Model II: clinical stage is excluded due to multicollinearity.
