# Supplementary material for: Retrospective Analysis of Prognostic Factors in 205 Patients with Laryngeal Squamous Cell Carcinoma Who Underwent Surgical Treatment
Source: PLoS One. 2013 Apr 4;8(4):e60157. doi: 10.1371/journal.pone.0060157 (PMC3617169; doi:10.1371/journal.pone.0060157)
Supplement: Table S3 — Clinical stages and locations. Advanced clinical stage was significantly associated with supraglottic cancers, with 75.8% vs. 39.2% having supraglottic and glottic cancers, respectively. Conversely, positive surgical margins were not significantly associated with either cancer. (DOC) [file pone.0060157.s003.doc]

Table S3. Clinical stages and locations.

|  |  | Supraglottic(n=62) | Glottic(n=143) | *p*-value |
| --- | --- | --- | --- | --- |
| Clinical stage | I | 2 (3.2%) | 58 (40.6%) | <0.001 |
|  | II | 13 (21.0%) | 29 (20.3%) |  |
|  | III | 11 (17.7%) | 28 (19.6%) |  |
|  | IV | 36 (58.1%) | 28 (19.6%) |  |
| Surgical margin | Positive | 11 (17.7%) | 20 (14.0%) | 0.527 |
|  | Negative | 51 (82.3%) | 123 (86.0%) |  |
